# Association of Childhood Intrafamilial Aggression and Childhood Peer Bullying With Adult Depressive Symptoms in China

**DOI:** 10.1001/jamanetworkopen.2020.12557

**Published:** 2020-08-04

**Authors:** Qing Wang

**Affiliations:** 1 School of Public Health, Cheeloo Collage of Medicine, Shandong University, Jinan, China; 2 Institute for Medical Dataology, Cheeloo Collage of Medicine, Shandong University, Jinan, China; 3 Pudong Institute for Health Development, Shanghai, China

## Abstract

**Importance:**

Childhood intrafamilial aggression, a series of aggressive behaviors inflicted by family members, and adulthood mental health are associated with childhood peer bullying (eg, in the neighborhood or in school). However, few studies have been able to identify the contribution of childhood peer bullying to the association between childhood intrafamilial aggression and adult depression.

**Objective:**

To examine the mediating role of childhood peer bullying in the association between childhood intrafamilial aggression and depression in adulthood.

**Design, Setting, and Participants:**

This population-based cross-sectional study used data from the China Health and Retirement Longitudinal Study (CHARLS). Middle-aged and older adults (≥45 years) participated in a CHARLS wave from July 1 to September 30, 2015, and the CHARLS life history survey from June 1 to December 31, 2014, with complete data. A 4-step mediation model with logistic regression was run to test the mediating role of peer bullying. Data analysis was performed from October 1 to 30, 2019.

**Exposures:**

Childhood intrafamilial aggression, including parental physical maltreatment and sibling aggression, and peer bullying.

**Main Outcomes and Measures:**

Depressive symptoms measured by a categorical variable derived from the Center for Epidemiologic Studies Depression Scale, based on a cutoff score of 12.

**Results:**

Among the 15 450 respondents (mean [SD] age, 59.5 [9.9] years; 7987 women [51.7%]), 4422 (28.6%) were exposed to parental physical maltreatment; 986 (6.4%), to sibling aggression; and 2504 (16.2%), to peer bullying in childhood. Respondents experiencing intrafamilial aggression were more likely to be bullied by peers (parental physical maltreatment odds ratio [OR], 2.53 [95% CI, 2.25-2.83]; sibling aggression OR, 3.05 [95% CI, 2.46-3.78]). Children with these adverse experiences were at a higher risk of adult depression symptoms (parental physical maltreatment OR, 1.28 [95% CI, 1.15-1.42]; sibling aggression OR, 1.40 [95% CI, 1.13-1.74]; peer bullying OR, 1.78 [95% CI, 1.56-2.01]). Peer bullying mediated 30% (95% CI, 19%-42%) of the association between childhood parental maltreatment and adult depression and 35% (95% CI, 15%-54%) of the association between sibling aggression and depression symptoms in adulthood.

**Conclusions and Relevance:**

This study found that being bullied by peers was a mediator of the association between childhood intrafamilial aggression and depression in adulthood. The findings have important implications to mitigate the effect of early-life stress and promote life-course mental health through dealing with childhood intrafamilial aggression and peer bullying experiences coordinately.

## Key Points

**Question:**

What is the contribution of childhood peer bullying to the association between intrafamilial aggression exposure and depression symptoms in adulthood?

**Findings:**

In this national cross-sectional study of 15 450 respondents 45 years or older in China, being bullied by peers in childhood was a mediator of the association between childhood intrafamilial aggression (eg, parental physical maltreatment and sibling aggression) and adulthood depression symptoms. The contribution of peer bullying to the association was approximately 30%.

**Meaning:**

These findings recommend a life-course and integrated mental health policy accounting for intrafamilial aggression and peer bullying in childhood.

## Teaser

This cross-sectional study examines the mediating role of childhood peer bullying in the association between childhood intrafamilial aggression and depression in Chinese adults.

## Introduction

Intrafamilial aggression and peer bullying in childhood are recognized as major social issues worldwide, conferring considerable risk for life-course mental health problems.^1,2,3^ A growing number of studies^4,5,6,7,8,9,10,11,12,13^ have established a robust association between earlier-life exposure to adverse experience (eg, parental physical maltreatment, sibling aggression, or peer bullying) and later-life psychological outcomes, such as anxiety, depression, self-harm, and attempt or completion of suicide. Although our knowledge of the consequences of adverse experiences is primarily based on studies performed in highly industrialized countries with societies considered to be individualistic, such as North America, Europe, and Australia, similar associations began to be found in more collectivistic or Confucian cultures, such as China, where harsh parenting (eg, “spare the rod and spoil the child”) and sibling hierarchical relationships (eg, older siblings get greater respect, but also take on the responsibility of providing care for younger siblings) are standard.^9,12^

In addition, dozens of studies provide support for the view that children experiencing intrafamilial aggression were at a higher risk for peer bullying.^3,14,15,16,17,18,19^ Given the pairwise association among childhood intrafamilial aggression, peer bullying, and adulthood mental health, the experience of childhood peer bullying could be a mediator in explaining the association between childhood intrafamilial aggression and adult mental health that has yet to be assessed.

Nevertheless, a recent study highlighted that childhood parental maltreatment and peer bullying exposure has an independent effect on young adults’ mental health, and bullying has a stronger effect on adult mental health in comparison with childhood maltreatment.^20^ Consistent with the finding, childhood intrafamilial aggression and peer bullying are dealt with by 2 different departments in some countries (eg, China). If childhood intrafamilial aggression is associated with childhood peer bullying and adult depressive symptoms, dealing with intrafamilial aggression and peer bullying independently may neglect the potential association between intrafamilial aggression and peer bullying.

To clarify the association of childhood intrafamilial aggression and peer bullying with depression at a later age and enhance the effectiveness of related policy, this study aims to quantify the mediating role of exposure to childhood peer bullying in understanding the association between childhood intrafamilial aggression and depression symptoms at a later age in China. Childhood intrafamilial aggression included parental physical maltreatment and sibling aggression in this study. The former refers to any act or series of acts of physical aggression by a parent or a caregiver that results in harm, potential for harm, or threat of harm to a child. The latter is similar but conducted by siblings and is often seen as a normative and harmless component of sibling relationships.^3,18^ This study measured sibling aggression, which is often neglected in research.

## Methods

### Data

This study used data from the 2015 wave of the China Health and Retirement Longitudinal Study (CHARLS 2015) combined with the CHARLS life history survey. CHARLS is a nationally representative sample of people 45 years and older that used stratified multistage cluster sampling, and the final samples fell within 150 counties of 28 provinces across China. The participant and his or her spouse (if present) were interviewed face-to-face in each household. Written inform consent was obtained from all participants. The institutional review board at Peking University approved the CHARLS survey. A more detailed description of the study design and sampling procedure can be found in the cohort profile of CHARLS.^21^ Our research involved secondary analysis of established data sets and was not subject to ethical approval or informed consent according to the London School of Economics and Political Science research ethics policy and procedures.^22^ This study follows the Strengthening the Reporting of Observational Studies in Epidemiology (STROBE) reporting guideline for cross-sectional studies.

The first CHARLS nationwide data were collected in 2011, covering an extensive range of information, such as demographic characteristics, socioeconomic status, health status, insurance, and use of health care services. The CHARLS cohort was followed up every 2 years, with previous respondents tracked and including a small share of new respondents. The second and third waves were conducted in 2013 and 2015, respectively. In this study, we used the CHARLS 2015 wave, conducted from July 1 to September 30, 2015. In addition, the CHARLS life history survey, conducted from June 1 to December 31, 2014, retrospectively collected the life history information of all live respondents in the previous waves (2011 and 2013). The data include residence and relocation history, childhood history, educational history, and so on.

Overall, 18 780 individuals participated in both the CHARLS 2015 wave and the CHARLS life history sample. After excluding 1796 participants who did not complete the Center for Epidemiologic Studies Depression Scale (CES-D) and 1534 participants with missing values, the complete sample with data on childhood adverse experience, adult depression, and confounders consisted of 15 450 participants (Figure). For a robust check, we conducted multiple imputation for the sample that had completed the CES-D (n = 16 984).

**Figure.**
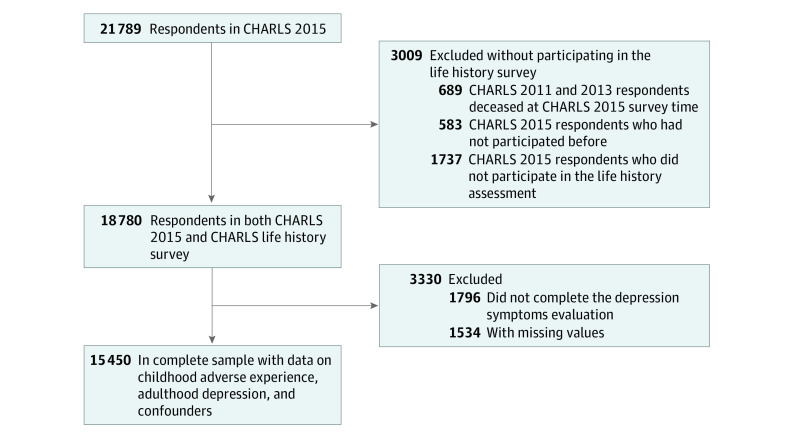
Study Flowchart Depressive symptoms were evaluated with the Center for Epidemiologic Studies Depression Scale. CHARLS indicates China Health and Retirement Longitudinal Study; CHARLS 2015, the 2015 wave of follow-up in CHARLS.

### Measures

#### Childhood Intrafamilial Aggression

Parental physical maltreatment was identified in response to the following questions: “When you were growing up, did your parents or guardian ever hit you? Was that often, sometimes, rarely, or never?” Following the rule of Chapman et al,^23^ participants were defined as experiencing physically adverse behavior by parents or guardians if they responded often or sometimes to the questions.

Sibling aggression was identified in a similar way with the questions, “When you were growing up, did your siblings ever hit you? Was that never, rarely, sometimes, or often?” Participants were defined as experiencing sibling aggression if they responded sometimes or often to the questions.^23^

#### Peer Bullying

Participants were defined as being bullied by peers as a child by the following questions^15^: “When you were a child, how often were you picked on or bullied by kids in your neighborhood (never, rarely, sometimes, or often)?” and “When you were a child, how often were you picked on or bullied by kids in your school (never, rarely, sometimes, or often)?” A response of often or sometimes to either question was defined as peer bullying.

#### Adult Depression Symptoms

A shortened modification of the CES-D scale including 7 items was used to measure depression symptoms.^24^ The items were evaluated as follows: (1) “was bothered by things,” (2) “had trouble keeping mind on tasks,” (3) “felt depressed,” (4) “felt everything he/she did was an effort,” (5) “felt fearful,” (6) “restless sleep,” and (7) “felt lonely.” The frequency in experiencing such symptoms in the previous week before the survey was encoded from 1 to 4, where 1 indicates none or rarely; 2, some or little; 3, occasionally or a moderate amount; and 4, most or all of the time. Summed scores ranged from 7 to 28, with higher scores indicating more depressive disorders and therefore worse mental health. A categorical variable for the CES-D score was created based on a usual cutoff score of 12.^25^ The variable equaled 1 if the CES-D score was at least 12, and 0 if otherwise. The validity and reliability of this shortened CES-D scale are supported by previous studies in China.^26,27^

### Statistical Analysis

Data analysis was performed from October 1 to 30, 2019. Descriptive statistics, including frequency with percentages for categorical variables in the entire sample and by sex, were reported. A logistic model was constructed to test the association between childhood intrafamilial aggression and depression symptoms at later age. Based on a conceptual framework of the World Health Organization, demographic characteristics, socioeconomic status, and level of physical health were controlled for the regression analysis.^28^ Adulthood socioeconomic status was measured by educational attainment, which was a binary measure for the upper secondary school level and above.

Childhood socioeconomic status was captured by both parents’ educational attainment and household financial status during the respondent’s childhood. For the father’s or mother’s educational attainment, upper secondary school or higher was encoded as 1; otherwise educational attainment was encoded as 0. In addition, respondents were asked to classify household financial status during their childhood period into 2 categories: worse than others or better than others.

Demographic variables included sex (reference group: female), marital status (reference group: married with spouse present, including common-law marriage; unmarried included single, divorced, or separated), and 65 years or older. Physical health was assessed by asking respondents if a physician had diagnosed any chronic disease. If the answer was “yes,” the variable was labeled as 1.

Odds ratios (ORs) and 95% CIs were reported for the logistic model. Weighted regression models with robust variance estimates were derived from generalized estimating equations to adjust the SEs for the stratified sampling design and response rate. The initial weight consisted of cross-sectional weights from CHARLS 2015. Then, the sample attrition adjustment method using the response propensity model was applied to obtain the weight of our sample.^29^ Because 1796 respondents did not complete the 7 questions of the CES-D scale, the final weights further accounted for the response probability of the CES-D question. The inverse probability weight factor is calculated by the inverse predicted probability of completing the assessment of CES-D for everyone.

As a robust check, the multiple imputation method was considered to impute missing data by creating 20 imputed data sets, and logistic models were then applied. The hypothesis that the association between childhood intrafamilial aggression and mental health at later age will be mediated by being bullied by peers was tested using a 4-step analysis with the Sobel approach.^30,31^ The method involves testing a direct path between childhood intrafamilial aggression and adult depression symptoms and then estimating how much the association is reduced by the inclusion of childhood peer bullying. Following Buis,^32^ total effects were calculated and decomposed into direct and indirect effects. The details of the analysis are presented in the eMethods in the Supplement.

Two-tailed *P* < .05 indicated statistical significance. STATA, version 14 (StataCorp LLC), was used for all calculations.

## Results

The mean (SD) age of the 15 450 respondents in the present study was 59.5 (9.9) years, with 7987 women (51.7%) and 7463 men (48.3%). The mean (SD) CES-D score was 12.2 (5.0), with 5954 (38.5%) reporting CES-D scores of at least 12. For childhood adverse experience, 4422 (28.6%) were exposed to parental physical maltreatment; 986 (6.4%), to sibling aggression; and 2504 (16.2%), to peer bullying (Table 1). The observed characteristics for participants with incomplete data are presented in eTable 1 in the Supplement.

**Table 1.** Participant Descriptive Statistics

Variable	Participants, No. (%)		
	All (N = 15 450)	Male (n = 7463)	Female (n = 7987)
CES-D score^a^			
<12	9496 (61.5)	5178 (69.4)	4318 (54.1)
≥12	5954 (38.5)	2285 (30.6)	3669 (45.9)
Childhood parental physical maltreatment			
No	11 028 (71.4)	4902 (65.7)	6126 (76.7)
Yes	4422 (28.6)	2561 (34.3)	1861 (23.3)
Childhood sibling aggression			
No	14 464 (93.6)	7011 (93.9)	7453 (93.3)
Yes	986 (6.4)	452 (6.1)	534 (6.7)
Childhood peer bullying			
No	12 946 (83.8)	6125 (82.1)	6821 (85.4)
Yes	2504 (16.2)	1338 (17.9)	1166 (14.6)
Marital status			
Married	13 597 (88.0)	6780 (90.8)	6817 (85.4)
Unmarried	1853 (12.0)	683 (9.2)	1170 (14.6)
Educational attainment			
Upper secondary school and above	13 767 (89.1)	6357 (85.2)	7410 (92.8)
Lower secondary school and below	1683 (10.9)	1106 (14.8)	577 (7.2)
Financial situation before 17 y of age			
Worse off than others	6064 (39.2)	3065 (41.1)	2999 (37.5)
Same as or better than others	9386 (60.8)	4398 (58.9)	4988 (62.5)
Parents’ educational attainment			
Upper secondary school and above	3910 (25.3)	1768 (23.7)	2143 (26.8)
Lower secondary school and below	11 540 (74.7)	5695 (76.3)	5844 (73.2)
Have chronic disease			
Yes	8214 (53.2)	3840 (51.5)	4374 (54.8)
No	7236 (46.8)	3623 (48.5)	3613 (45.2)
Sex			
Female	7987 (51.7)	NA	7987 (100)
Male	7463 (48.3)	7463 (100)	NA
Age, y			
≥65	4707 (30.5)	2486 (33.3)	2221 (27.8)
<65	10 743 (69.5)	4977 (66.7)	5766 (72.2)

Abbreviations: CES-D, Center for Epidemiologic Studies Depression Scale; NA, not applicable.

^a^ A categorical variable for CES-D was created based on a usual cutoff score of 12, which was used to measure depression symptoms.

Individuals who experienced intrafamilial aggression were more likely to be bullied by peers (parental physical maltreatment odds ratio [OR], 2.53 [95% CI, 2.25-2.83]; sibling aggression OR, 3.05 [95% CI, 2.46-3.78]) (eTable 2 in the Supplement). Positive associations among childhood intrafamilial aggression, peer bullying, and depression symptoms in adulthood are presented in Table 2. Compared with respondents who did not experience intrafamilial aggression in childhood, those who did were more likely to have depression symptoms (childhood parental physical maltreatment OR, 1.28 [95% CI, 1.15-1.42]; childhood sibling aggression OR, 1.40 [95% CI, 1.13-1.74]; childhood peer bullying OR, 1.78 [95% CI, 1.56-2.01]). Judging from the ORs and their 95% CIs, which have very little overlap, the effect size of peer bullying was larger for depression than being financially worse off than others in childhood (OR, 1.33 [95% CI, 1.22-1.46]) and similar to having a current chronic illness (OR, 1.76 [95% CI, 1.61-1.92]).

**Table 2.** Association of Childhood Intrafamilial Aggression, Childhood Peer Bullying, and Adult Depressive Symptoms in China: Logistic Model^a^

Covariate	Effect size for association, OR (95% CI)^a^				
	Between childhood peer bullying and adult depression	Between childhood parental maltreatment and adult depression	Among childhood parental maltreatment, peer bullying, and adult depression	Between childhood sibling aggression and adult depression	Among childhood sibling aggression, peer bullying, and adult depression
Childhood peer bullying	1.78 (1.56-2.01)	NA	1.71 (1.50-1.95)	NA	1.74 (1.53-1.97)
Childhood intrafamilial aggression					
Childhood parental maltreatment	NA	1.28 (1.15-1.42)	1.19 (1.07-1.33)	NA	NA
Childhood sibling aggression	NA	NA	NA	1.40 (1.13-1.74)	1.26 (1.01-1.58)
Married	0.65 (0.57-0.74)	0.66 (0.58-0.75)	0.65 (0.57-0.74)	0.66 (0.57-0.75)	0.65 (0.57-0.74)
Educational attainment of upper secondary school or higher	0.59 (0.49-0.71)	0.61 (0.50-0.73)	0.60 (0.50-0.72)	0.60 (0.50-0.73)	0.59 (0.49-0.71)
Childhood financial situation worse off than others’	1.33 (1.22-1.46)	1.39 (1.27-1.52)	1.32 (1.21-1.45)	1.40 (1.28-1.53)	1.33 (1.22-1.46)
Parents’ educational attainment of upper secondary school or higher	0.95 (0.86-1.06)	0.94 (0.84-1.05)	0.94 (0.85-1.05)	0.95 (0.86-1.06)	0.95 (0.86-1.06)
Female	1.82 (1.66-2.00)	1.83 (1.67-2.01)	1.86 (1.70-2.04)	1.78 (1.62-1.95)	1.82 (1.66-2.00)
Aged ≥65 y	0.98 (0.89-1.09)	0.96 (0.87-1.06)	0.99 (0.90-1.10)	0.95 (0.86-1.05)	0.998 (0.89-1.09)
Having chronic diseases	1.76 (1.60-1.92)	1.76 (1.61-1.92)	1.76 (1.60-1.92)	1.76 (1.61-1.93)	1.76 (1.60-1.92)

Abbreviations: NA, not applicable; OR, odds ratio.

^a^ Includes 15 450 observations for each effect size.

After adjusting for peer bullying, intrafamilial aggression was still associated with a higher risk for depression, but the association was weakened by the inclusion of being bullied by peers in the childhood period (childhood parental maltreatment OR, 1.19 [95% CI, 1.07-1.33]; childhood sibling aggression OR, 1.26 [95% CI, 1.01-1.58]). When incomplete data were imputed with the multiple imputation method, the results were consistent (eTables 2 and 3 in the Supplement).

Table 3 presents the indirect contribution of being bullied by peers in the association of childhood intrafamilial aggression and adulthood mental health. Childhood peer bullying was a mediator of this association. It explained the association in part; the contribution of peer bullying was 30% (95% CI, 19%-42%) of the association between childhood parental maltreatment and adult depression symptoms and 35% (95% CI, 15%-54%) of the association between sibling aggression and adult depression symptoms.

**Table 3.** Mediation of Childhood Peer Bullying in the Association Between Childhood Intrafamilial Aggression and Adult Depression Symptoms

Mediation of childhood peer bullying^a^	Association of childhood parental maltreatment and adult depression symptoms	Association of sibling aggression and adult depression symptoms
Effect, OR (95% CI)		
Indirect	1.08 (1.07-1.10)	1.12 (1.09-1.15)
Direct	1.21 (1.13-1.29)	1.23 (1.09-1.42)
Total	1.31 (1.22-1.40)	1.39 (1.21-1.59)
Proportion of indirect to total effect, % (95% CI)	30 (19-42)	35 (15-54)

Abbreviation: OR, odds ratio.

^a^ The Sobel test found childhood peer bullying was a mediator in the association of childhood intrafamilial aggression with adult depressive symptoms, with a *z* value of 6.89 and 6.77, respectively.

## Discussion

In this large, population-based, cross-sectional study, we found that exposure to intrafamilial aggression or peer bullying during childhood was associated with adult risk of depression in China, and peer bullying had a closer association with life-course depression, with a similar effect size of having a chronic disease. Our results are similar to those of previous studies in western countries^4,5,6,7,8,9,10,11,12,13,20,33,34,35^ that have found a negative association between childhood adverse experience and adult mental health, despite culture differences. This study adds to the emerging evidence that sibling aggression, a less-studied type of intrafamilial aggression, was also associated with depression symptoms in adulthood.^3^

As expected, analyses indicated that the association of childhood intrafamilial aggression and adult depression was partially mediated by being bullied by peers. Childhood intrafamilial aggression was associated with elevated levels of depressive symptoms at a later age through increased likelihood of peer bullying. The results were consistent with those of previous research, in which peer bullying was found to be associated with prior experiences and subsequent mental health problems.^35,36,37,38^ Furthermore, a study showed the similar view that the association between maltreatment and depression at the same stage was mediated by peer bullying among US children from low socioeconomic backgrounds.^17^

The mediational findings could be explained by organizational theories of development suggesting continuity in relationships.^38,39^ Experience of intrafamilial aggression may lead children to develop negative expectations pertaining to themselves and others and a concept of relationships involving bullying and agression.^40^ In subsequent relationships, these children may continuously recreate familiar social environments to validate their expectations so that they could maintain a coherent sense of self, which may help them adapt to maltreated and neglected homes.^41^ In this case, children experiencing intrafamilial aggression might be vulnerable to bullying behaviors, which may put them at risk for mental health issues at an older age.

To our knowledge, this study is the first to establish the mediating role of childhood peer bullying in the association between adult mental health and childhood exposure to intrafamilial aggression, including parental physical maltreatment and particularly sibling aggression. The present study is unique in demonstrating to what extent childhood exposure to intrafamilial aggression is associated with adulthood mental health through peer bullying exposure.

Our investigation sheds light on the creation and implementation of prevention and intervention programs to mitigate the effect of early-life stress and to promote life-course mental health. First, to pursue the premise of maximizing mental health throughout one’s life, a life-course policy on health promotion should be adopted instead of only targeting the mental health of specific age groups. The possibility that the influences of childhood intrafamilial aggression extend to health in later age implies that policy interventions should work throughout the entire life cycle, beginning from childhood. Those growing up within an environment of intrafamilial aggression may not only be vulnerable to poor mental health but may also experience peer bullying; thus, mental health interventions or policies should be tailored to focus on these people, recognizing early warning signs of parental physical maltreatment and in particular sibling aggression.

Second, antibullying efforts at school should also account for children’s parent and sibling relationships. Because peer bullying is a mediator of the association between intrafamilial aggression and mental health in later age, it is important for schools, health services, and other agencies to coordinate their responses to intrafamilial aggression and peer bullying.

### Limitations

When we studied childhood adverse experiences, our measurements were crude owing to data constraints of CHARLS. The effects of onset and severity of childhood adverse experience were not investigated in this study and should be in future studies. In addition, the indicators to measure childhood adverse experience were retrospective self-evaluation with potential measurement error issues, whereas prospective evidence suggests that effects of childhood adverse experience reach this far.^4,7,42^ However, self-evaluation could reveal participants’ own perceptions of their internal states and has been found to be congruent with peer evaluation.^43,44^ Finally, girls are subjected more to relational bullying (eg, spreading rumors or social exclusion), which may lead to adulthood mental health issues, but it has not been assessed in this study.^11,18,45^

## Conclusions

In this cross-sectional study, exposure to intrafamilial aggression (eg, parental physical maltreatment and sibling aggression) and peer bullying was associated with depression in adulthood, and peer bullying was more strongly associated with depression symptoms at later age. Furthermore, the association between childhood intrafamilial aggression and adult depression symptoms was mediated by peer bullying. In this case, the life-course policy on mental health promotion should be designed and implemented to take childhood adverse experience into account and attach more importance to peer bullying.

## Notes

Supplement. **eMethods.** A 4-Step Regression Analysis to Test the Mediator of Childhood Peer Bullying **eTable 1.** Descriptive Statistics for Respondents With Incomplete Data **eTable 2.** Association Between Childhood Intrafamilial Aggression and Childhood Peer Bullying: Logistic Model **eTable 3.** Association of Childhood Intrafamilial and Childhood Peer Bullying With Adult Depressive Symptoms in China With Missing Data Imputed: Logistic Model
