# Supplementary material for: Association of Childhood Intrafamilial Aggression and Childhood Peer Bullying With Adult Depressive Symptoms in China
Source: JAMA Netw Open. 2020 Aug 4;3(8):e2012557. doi: 10.1001/jamanetworkopen.2020.12557 (PMC7403920; doi:10.1001/jamanetworkopen.2020.12557)
Supplement: Supplement. — eMethods. A 4-Step Regression Analysis to Test the Mediator of Childhood Peer Bullying eTable 1. Descriptive Statistics for Respondents With Incomplete Data eTable 2. Association Between Childhood Intrafamilial Aggression and Childhood Peer Bullying: Logistic Model eTable 3. Association of Childhood Intrafamilial and Childhood Peer Bullying With Adult Depressive Symptoms in China With Missing Data Imputed: Logistic Model [file jamanetwopen-3-e2012557-s001.pdf]

Supplementary Online Content

Wang Q. Association of childhood intrafamilial aggression and childhood peer bullying with adult depressive symptoms in China. *JAMA Netw Open*. 2020;3(8):e2012557. doi:10.1001/jamanetworkopen.2020.12557

eMethods. A 4-Step Regression Analysis to Test the Mediator of Childhood Peer Bullying

eTable 1. Descriptive Statistics for Respondents With Incomplete Data

eTable 2. Association Between Childhood Intrafamilial Aggression and Childhood Peer Bullying: Logistic Model

eTable 3. Association of Childhood Intrafamilial and Childhood Peer Bullying With Adult Depressive Symptoms in China With Missing Data Imputed: Logistic Model

This supplementary material has been provided by the authors to give readers additional information about their work.

©2020 Wang Q. *JAMA Network Open*.

eMethods. A 4-Step Regression Analysis to Test the Mediator of Childhood Peer Bullying

The hypothesis that the association between childhood intra-familial aggression and depression symptoms at later age will be mediated by peer bullying was tested using a four-step analysis with Sobel approach. [1,2] Specifically, we tested the direct path between childhood physical maltreatment by guardian or siblings and adulthood depression symptoms, and estimated the extent to which the association was weakened by the inclusion of peer bullying exposure in childhood period. This analysis addresses the following questions:

Step 1: estimating the strength of the direct path. a Logistic model was constructed to test whether childhood intra-familial aggression was significantly associated depression symptoms at later age without adjusting for childhood peer bullying victimization.

$$P(H_i = 1) = c_1 + c_2 * mal_i + c_3 * CON_i$$

Where, H_i=1 indicates respondents having depression symptoms in adulthood; mal_i measures childhood parental physical maltreatment or sibling aggression victimization, CON_i are demographic characteristics, socioeconomic status, and level of physical health. c_1, c_2, c_3 are the coefficients to measure. If childhood intra-familial aggression was significantly associated depression symptoms at later age, the mediator of childhood peer bullying victimization in the association of childhood intra-familial aggression and depression symptoms at later age was then tested.

Step 2: a Logistic model was applied to estimate whether childhood intra-familial aggression was significantly related to childhood peer bullying victimization.

$$P(bully_i = 1) = a_1 + a_2 * mal_i + a_3 * CON_i$$

Where, $bully_i$ presents childhood peer bullying exposure, and a_1, a_2, a_3 are the coefficients to measure.

Collect the parameter estimate a_2, and its standard error, s_a.

Step 3: a Logistic model to estimate the association between childhood intra-familial aggression and adulthood depression symptoms upon the addition of childhood peer bullying to the model.

$$P(H_i = 1) = b_1 + b_2 * bully_i + b_3 * mal_i + b_3 * CON_i$$

Where, $b_1, b_2, b_3$are the coefficients to measure.

Collect the parameter estimate b_2, and its standard error, s_b.

Using the coefficient estimates a_2 and b_2 and their standard errors s_a and $\mathrm{s}_\mathrm{b}$from step 2 and 3，

compute the standardized elements: $\mathrm{Z}_\mathrm{a} = \frac{\mathrm{a}_2}{\mathrm{s}_\mathrm{a}}$, $\mathrm{Z}_\mathrm{b} = \frac{\mathrm{b}_2}{\mathrm{s}_\mathrm{b}}$,

their product: $\mathrm{Z}_{\mathrm{a}*\mathrm{b}} = \mathrm{Z}_\mathrm{a} * \mathrm{Z}_\mathrm{b}$, and their collected standard error $\sqrt{1 + {\mathrm{Z}_\mathrm{a}}^2 + {\mathrm{Z}_\mathrm{b}}^2}$

Step 4: compute the z-test that combines results from *step 2 and 3*, to indicate whether there is a significant mediation effect:

$$\mathrm{Z}_{\mathrm{med}} = \frac{\mathrm{Z}_{\mathrm{a}*\mathrm{b}}}{\sqrt{1 + {\mathrm{Z}_\mathrm{a}}^2 + {\mathrm{Z}_\mathrm{b}}^2}}$$

Test $\mathrm{Z}_{\mathrm{med}}$ was significant at the p=0.05 level if it exceeds |1.96| (for a 2-tailed test with p=0.05), peer bullying was regarded as a mediator of the association between childhood intra-familial aggression and depression symptoms at later age.

©2020 Wang Q. *JAMA Network Open*.

Then, following Buis, total effects were calculated in logistic regression, and the effects decomposed into direct and indirect effects.[3] The use of the bootstrap was proposed in order to estimate standard errors.

1. Iacobucci D. Mediation analysis and categorical variables: the final frontier. J Consum Psychol. 2012; 22: 582–594.
2. VanderWeele TJ. Mediation analysis: a practitioner's guide. Annu R Publ Health. 2016; 37: 17-32.
3.Buis M L. Direct and Indirect Effects in a Logit Model. The Stata Journal: Promoting communications on statistics and Stata, 2010, 10(1):11-29.

©2020 Wang Q. *JAMA Network Open*.

eTable 1. Descriptive Statistics for Respondents With Incomplete Data

	All [a]		Male		Female	
**Variable**	**Obs.**	**Percent(%)**	**Obs.**	**Percent(%)**	**Obs.**	**Percent(%)**
**Depression**	18,780	100	8,705	100	9,754	100
CES-D<12 [b]	10,454	55.7	5,562	63.9	4,724	48.4
CES-D>=12	6,530	34.7	2,452	28.2	4,012	41.1
Unknown	1,796	9.6	691	7.9	1,018	10.4
**Childhood parental physical maltreatment**	17,941	100	8,516	100	9,425	100
No	12,922	72.0	5,647	66.3	7,275	77.2
Yes	5,019	28.0	2,869	33.7	2,150	22.8
**Childhood sibling aggression**	17,941	100	8,516	100	9,425	100
No	16,796	93.6	8,003	94.0	8,793	93.3
Yes	1,145	6.4	513	6.0	632	6.7
**Childhood peer bullying victimization**	18,288	100	8,497	100	9,487	100
No	15,310	83.7	6,963	82.0	8,092	85.3
Yes	2,978	16.3	1,534	18.0	1,397	14.7
**Marital status**	18,759	100	8,703	100	9,754	100
Married	16,252	86.6	7,847	90.2	8,121	83.3
Unmarried	2,507	13.4	856	9.8	1,633	16.7
**Education**	18,780	100	8,705	100	9.754	100
Upper secondary and above	16,914	90.1	7,509	86.2	9,121	93.5
Lower secondary and below	1,866	9.9	1,199	13.8	633	6.5
**Financial situation before age 17**	18,555	100	8,622	100	12,349	100
Worse off than Them	7,317	39.4	3,587	41.6	3,611	37.5
Same or better than them	11,238	60.6	5,035	58.4	6,015	62.5

©2020 Wang Q. *JAMA Network Open*.

**Parents’ education**	18,780	100	8,705	100	9,754	100
Upper secondary and above	4,847	25.8	2,210	24.4	2,629	27.0
Lower secondary and below	13,933	74.2	6,585	75.7	7,125	73.0
**Having chronic disease**	18,723	100	8,705	100	9,754	100
Yes	9,729	52.0	4,356	50.0	5,262	46.0
No	8,994	48.0	4,349	50.0	4,492	54.0
**Sex**	18,459	100	--	--	--	--
Female	9,754	52.8	--	--	9,754	100
Male	8,705	47.2	8,705	100	--	--
**Aged people**	18,231	100	8,516	100	9,425	100
>=65	5,783	31.7	2,906	34.1	2,824	30.0
<65	12,448	68.3	5,610	65.9	6,601	70.0

[a] Due to missing value for sex, the sum of males and females is fewer than the total sample size.

[b] The Center for Epidemiologic Studies Depression: CES-D

©2020 Wang Q. *JAMA Network Open*.

eTable 2. Association Between Childhood Intrafamilial Aggression and Childhood Peer Bullying: Logistic Model

	Excluding missing data		Missing data imputed [a]	
	**OR [b] (95%CI)**	**OR (95%CI)**	**OR (95%CI)**	**OR (95%CI)**
**Childhood intra-familial aggression**				
**Childhood parental maltreatment**	2.53		1.67	
	(2.25 - 2.83)		(1.55 - 1.80)	
**Childhood sibling aggression**		3.05		2.42
		(2.46 - 3.78)		(2.14 - 2.73)
**Married**	0.95	0.93	0.64	0.64
	(0.80 - 1.13)	(0.78 - 1.10)	(0.58 - 0.71)	(0.58 - 0.71)
**Senior high school and above**	1.28	1.23	1.02	1.02
	(1.05 - 1.56)	(1.01 - 1.49)	(0.91 - 1.15)	(0.90 - 1.14)
**Children financial: worse off than Them**	2.13	2.18	1.88	1.89
	(1.91 - 2.39)	(1.96 - 2.44)	(1.74 - 2.02)	(1.76 - 2.04)
**Parents' high school and above**	1.02	1.08	1.69	1.70
	(0.90 - 1.17)	(0.94 - 1.23)	(1.57 - 1.83)	(1.57 - 1.83)
**Female**	0.86	0.75	0.89	0.84
	(0.77 - 0.96)	(0.67 - 0.83)	(0.83 - 0.97)	(0.77 - 0.91)
**Aged people**	0.61	0.59	0.89	0.89
	(0.54 - 0.70)	(0.52 - 0.68)	(0.82 - 0.97)	(0.81 - 0.97)
**Having chronic diseases**	1.06	1.07	0.99	1.00
	(0.95 - 1.19)	(0.95 - 1.19)	(0.91 - 1.07)	(0.92 - 1.08)
Observations	15,450	15,450	16,984	16,984

[a] Multiple imputation methods to impute missing data

[b] Odd Ratio: OR; CI: Confidence Interval.

©2020 Wang Q. *JAMA Network Open*.

eTable 3. Association of Childhood Intrafamilial and Childhood Peer Bullying With Adult Depressive Symptoms in China With Missing Data Imputed: Logistic Model[a]

	Effect size, OR (95%CI) [b]				
	Association between childhood peer bullying and adulthood depression	Association between childhood parental maltreat and adulthood depression	Association among childhood parental maltreat, peer bullying and adulthood depression	Association between childhood sibling agression and adulthood depression	Association among childhood sibling agression, peer bullying and adulthood depression
**Childhood peer bullying**			1.72	1.65	1.68
			(1.57 - 1.87)	(1.51 - 1.81)	(1.54 - 1.84)
**Childhood parental maltreatment**	1.29			1.20	
	(1.20 - 1.38)			(1.12 - 1.29)	
**Childhood sibling aggression**		1.38			1.24
		(1.21 - 1.56)			(1.09 - 1.41)
**Married**	0.65	1.38	0.65	0.65	0.65
	(0.58 - 0.71)	(1.21 - 1.56)	(0.59 - 0.72)	(0.59 - 0.72)	(0.59 - 0.72)
**Senior high school and above**	0.58	1.38	0.57	0.57	0.57
	(0.52 - 0.65)	(1.21 - 1.56)	(0.51 - 0.64)	(0.51 - 0.64)	(0.51 - 0.64)
**Children financial: worse off than Them**	1.46	1.38	1.40	1.39	1.40
	(1.37 - 1.56)	(1.21 - 1.56)	(1.31 - 1.50)	(1.30 - 1.49)	(1.31 - 1.50)
**Parents' high school and above**	0.97	1.38	0.97	0.96	0.97
	(0.90 - 1.04)	(1.21 - 1.56)	(0.90 - 1.04)	(0.89 - 1.04)	(0.90 - 1.04)
**Female**	1.91	1.38	1.89	1.93	1.89
	(1.78 - 2.04)	(1.21 - 1.56)	(1.77 - 2.02)	(1.81 - 2.07)	(1.77 - 2.02)

©2020 Wang Q. *JAMA Network Open*.

**Aged people**	1.02	1.38	1.04	1.05	1.04
	(0.94 - 1.10)	(1.21 - 1.56)	(0.96 - 1.12)	(0.97 - 1.13)	(0.97 - 1.13)
**Having chronic diseases**	1.73	1.38	1.75	1.74	1.75
	(1.62 - 1.85)	(1.21 - 1.56)	(1.63 - 1.87)	(1.63 - 1.86)	(1.63 - 1.87)
Observations	16,984	16,984	16,984	16,984	16,984

[a] Multiple imputation methods to impute missing data

[b] Odd Ratio: OR; CI: Confidence Interval.

©2020 Wang Q. *JAMA Network Open*.
